# Safety and Efficacy of Heparinization During Mechanical Thrombectomy in Acute Ischemic Stroke

**DOI:** 10.3389/fneur.2019.00299

**Published:** 2019-03-29

**Authors:** Ming Yang, Xiaochuan Huo, Feng Gao, Anxin Wang, Ning Ma, David S. Liebeskind, Yongjun Wang, Zhongrong Miao

**Affiliations:** ^1^Department of Neurology, Capital Medical University, Beijing, China; ^2^Interventional Neuroradiology, Beijing Tiantan Hospital, Capital Medical University, Beijing, China; ^3^China National Clinical Research Center for Neurological Diseases, Beijing, China; ^4^Center of Stroke, Beijing Institute for Brain Disorders, Beijing, China; ^5^Neurovascular Imaging Research Core and Department of Neurology, UCLA Stroke Center, University of California, Los Angeles, Los Angeles, CA, United States

**Keywords:** mechanical thrombectomy, heparinization, acute ischemic stroke, symptomatic intracerebral hemorrhage (sICH), distal embolization, functional outcomes

## Abstract

**Background:** The benefits of heparization during mechanical thrombectomy (MT) with newer generation thrombectomy devices, and if it is counterbalanced by the increased risk of intracranial hemorrhage (ICH) remain unknown.

**Methods:** We included eligible patients who underwent MT from the ANGEL registry study (2015-2017) in China. Subjects in the current analysis were dichotomized into two groups according to whether adequate heparinization during MT was performed. In the heparinization group, unfractionated heparin was infused at 50–100 IU/Kg at first and additional 1,000 IU at intervals of an hour during the operation. Safety outcomes (symptomatic intracerebral hemorrhage [sICH], ICH and distal embolization) and efficacy outcomes (artery recanalization and functional outcomes at 3-month follow-up) were compared between groups.

**Results:** We included 619 patients from the entire cohort of 917 patients. The average age of them was 63.9 ± 13.7 years, 269 (43.5%) were treated with heparinization during MT. Heparinization during MT didn't significantly influence recanalization rates, total ICH and long-term mortality (adjusted *p* > 0.05 for all). But sICH and distal embolization occurred more frequently (9.3 vs. 5.1%, adjusted *p* = 0.02; 7.1 vs. 3.1%, adjusted *p* = 0.04, respectively), while functional independence appeared less likely (39.8 vs. 47.4%, adjusted *p* = 0.01) in heparinization group than that in non-heparinization group. Multivariable logistic regression analyses showed that heparinization during MT was an independent predictor for sICH (Odds ratio 2.36 [1.19–4.67], *p* = 0.01) in addition to cardio-embolism stroke and posterior circulation stroke (PCS), and an independent predictor for poor outcome (Odds ratio 1.79 [1.23–2.59], *p* < 0.01) besides age, bridging intravenous thrombolysis, admission NIHSS, drinking and PCS.

**Conclusion:** Heparinization during MT may be associated with increased risk of safety outcomes over sICH and distal embolization, as well as efficacy outcomes over long-term poor outcome. Further randomized controlled trials are needed.

## Introduction

Conventional systemic heparinization during neurointerventional procedures has demonstrated benefits against thromboembolic complications. A standard systemic heparinization for maintaining activated clotting time between 250 and 300 s was widely suggested during percutaneous transluminal intracranial angioplasty and stenting (1). However, few studies emphasized on the safety and efficacy profile of systemic heparinization during mechanical thrombectomy (MT) for acute ischemic stroke (AIS). Theoretically, heparinization during MT may be of additional therapeutic effects by preventing microthrombus formation and restoring microvascular blood perfusion (2), but the potential risk of hemorrhagic complications must be taken into consideration.

Several *post-hoc* analysis of earlier endovascular trials (Multi MERCI and TREVO 2) with old thrombectomy processes suggested the relative safety of periprocedural heparin use (3–5). Given the small simple size, heterogeneity of treatment protocols and devices, their practical implications were limited. In addition, substantial difference exists in the usage of periprocedural heparin across different hospitals. Without exception, recent landmark randomized controlled clinical trials on MT for AIS conducted heparinization during MT according to local management protocols (6–8). Thus, the benefits of heparization during MT with newer generation thrombectomy devices, and if it's counterbalanced by the increased risk of intracranial hemorrhage (ICH) remain unknown. The objective of this study was to evaluate the safety and efficacy profile of heparinization during MT for acute ischemic stroke in the Acute Ischemic Stroke Cooperation Group of Endovascular Treatment (ANGEL) registry.

## Methods

### Patient Enrollment

We included eligible patients from the ANGEL registry study. ANGEL is a multi-centric, nationwide, prospective registry study launched in June 2015 and terminated in December 2017, aiming to evaluate MT delivery and to improve MT algorithm in clinical practice for AIS patients in China. It included 20 comprehensive stroke centers, which performed over 15 MT cases every year. Consecutive AIS patients owing to proximal cerebral major artery occlusion were included according to the following criteria: (1) age ≥ 18 years; (2) modified Rankin Scale (mRS) prior to the index stroke ≤ 1; (3) onset to puncture (OTP) time <12 h for anterior circulation stroke (ACS) and <24 h for posterior circulation stroke (PCS); (4) computed tomographic angiography (CTA), magnetic resonance angiography (MRA) or digital subtraction angiography (DSA) was performed when clinical symptoms severity suggested proximal artery occlusion, including intracranial internal carotid artery (ICA), M1/M2 segment of middle cerebral artery (MCA), basilar artery (BA) occlusions, or dominant vertebral artery (VA) occlusions (functional basilar occlusions). Tandem extracranial ICA and intracranial large artery occlusions were also included.

Excluding patients with NIHSS score <6, intra-arterial thrombolysis alone, thrombocytopenia, coagulation factor deficiency, inadequate heparinization for lower-dose of intravenous heparin, MT patients were dichotomized into heparinization group and non-heparinization group according to whether adequate heparinization during MT was adopted. All candidates underwent emergency head non-contrast computed tomography (CT) before treatment. For patients within 4.5 h from stroke onset, intravenous thrombolysis (IVT) was conducted while preparing for MT (Direct bridging IVT therapy) according to current guidelines (9). And MT was directly performed directly if IVT was contraindicated or refused. In cases with unknown or prolonged (>6 h) time window, DWI/PWI or CBV/CBF mismatch was suggested to define demonstrate infarct core/penumbra mismatch for patient selection for the mechanical recanalization.

The protocol and data collection of the ANGEL study were approved by the ethics committee of Beijing Tiantan Hospital and all other participating centers. And all participants or their representatives provided a written informed consent before inclusion into the study.

### Baseline Data Collection

Baseline characteristics on demographics, medical history, vascular risk factors, time interval from onset to groin puncture (OTP) and recanalization (OTR), initial National Institutes of Health Stroke Scale (NIHSS) score, baseline Alberta Stroke Program Early CT Score (ASPECTS) according to pretreatment non-contrast CT (for anterior circulation stroke), etiology according to the Trial of ORG 10172 in Acute Stroke Treatment (TOAST) classification (10), and intraprocedural details were all collected and sent to the central core laboratory in digital forms by trained coordinators.

All subjects underwent CT or MRI scanning after 24 h post-MT or whenever an ICH was suspected. All imaging data, including head CT and CTA, MRI and MRA, DSA images during the endovascular therapy, and follow-up CT or MRI imaging were anonymized and reviewed centrally by two independent readers (M.Y. and X.C.H) who were blind to subjects' baseline information. Disagreements were resolved by consensus or by the judgment of an otherwise third reader.

### Endovascular Treatment

All eligible patients underwent MT immediately after indication assessment according to current guidelines of endovascular treatment (9). All subjects who underwent MT employed stent retriever (Solitaire AB/FR, Covidien/ev3, Irvine, CA; Trevo Proview, Stryker, CA), aspiration device (Penumbra, Alameda, CA) as the first recanalization option according to protocol. For MT failed cases, additional thrombectomy attempts and alternative rescue therapies were adopted at the discretion of the operator both for the heparinization and non-heparinization group, including intra-arterial/intravenous tirofiban administration, intra-arterial thrombolysis, balloon angioplasty, and emergent stenting.

Heparinization during MT was performed empirically per individual operator's discretion according to local protocols. Heparinization was defined as intravenous administration of unfractionated heparin, being infused at 50–100 IU/Kg at first and additional 1,000 IU at intervals of an hour during the operation (11, 12). Oral antiplatelet therapy (aspirin100 mg or clopidogrel 75 mg once daily) or dual antiplatelet therapy were given according to head CT 24 h post-MT as a routine according to local protocols. All intraprocedural details were digitally documented for further analysis.

### Safety and Efficacy Outcomes

The primary safety endpoints were sICH evaluated on CT or T2^*^MR images within post-MT 24 h. sICH was defined according to ECASS-III (defined as worsening ≥ 4 points in NIHSS and associated with ICH) (13). The secondary safety outcome was any ICH on follow-up CT or T2^*^MR images and distal embolization during MT. Distal embolization are procedure-related complications that filling defects distal to the occlusion presented on DSA after the retrieval or aspiration, but absent on preceding CTA/MRA or the initial DSA were considered as distal embolization as previous reported (14). The long-term functional outcome was assessed with mRS (range 0~6) at 3-month follow-up by trained research coordinators who were blinded to subjects' baseline characters. The primary efficacy endpoints were long-term functional independence at 3-month follow-up (defined as mRS ≤2). The secondary efficacy outcome was mortality (mRS = 6), poor outcome (mRS 3~5) at 3-month follow-up and successful artery recanalization, which was defined as a modified Tissue Thrombolysis in Cerebral Ischemia (mTICI) grade ≥ 2b on the final angiogram during MT.

### Statistical Analysis

Baseline characteristics, all safety and efficacy outcomes including sICH, total ICH, distal embolization, artery recanalization, and long-term functional outcome were compared between heparinization group and non-heparinization group. We used χ^2^-test for categorical variables, one-way analysis of variance or Kruskal-Wallis test for continuous variables. We fitted logistic regression model to explore the correlation between primary/secondary endpoints and heparinization after adjusted for potential confounding factors based on theoretical considerations and baseline characteristic statistical differences by univariate analysis (including demographics, vascular disease risk factors, intraprocedural manipulations, and pathogenesis of stroke). Besides, we further conducted univariable and multivariable logistic regression analyses to assess the associations of heparinization and other baseline characteristics with the probability of sICH and poor outcome. Cut-off of *P* < 0.1 in univariable logistic regression analysis was used for selection of candidate variables for inclusion in multivariable logistic regression models.

All statistical analyses were conducted with SAS software version 9.4 (SAS Institute Inc., Cary, NC). Two-tailed *P* < 0.05 were considered as statistically significant.

## Results

### Demographics and Baseline Characteristics

**Figure 1:**
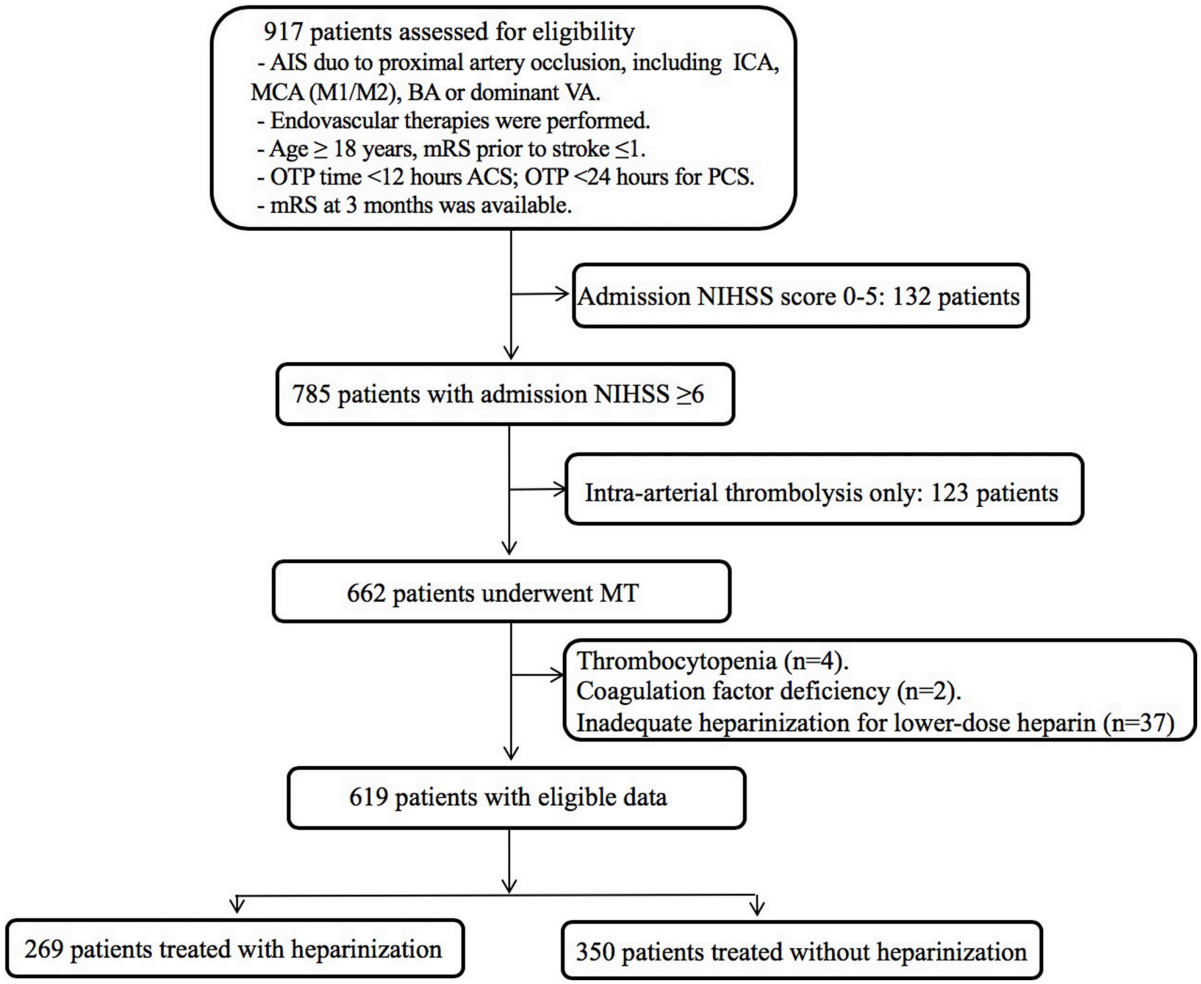
Flow chart of patient inclusion steps and findings analyzed at each step. AIS, acute ischemic stroke; ICA, internal carotid artery; MCA, middle cerebral artery; BA, basilar artery; VA, vertebral artery; mRS, modified Rankin Scale; OTP time, onset to puncture time; OTR time, onset to recanalization time; ACS, anterior circulation stroke; PCS, posterior circulation stroke; NIHSS, National Institutes of Health Stroke Scale score; MT, mechanical thrombectomy.

A total of 917 patients who underwent endovascular treatment were recruited in the ANGEL registry study from June 2015 to December 2017. Finally, 619 patients underwent MT were included in this analysis after excluding for admission NIHSS score <6 (*n* = 132), intra-arterial thrombolysis alone (*n* = 123), thrombocytopenia (*n* = 4), coagulation factor deficiency (*n* = 2), inadequate heparinization for lower-dose of intravenous heparin (*n* = 37) (Figure 1). Baseline clinical characteristics and intraprocedural options are shown in Table 1. Of them, 269 (43.5%) were treated with heparinization during MT, the average age was 63.9 ± 13.7 years and 404 (65.3%) were male. Three hundred and ninety two (63.3%) patients underwent rescue therapy, including 90 (14.5%) patients with balloon angioplasty, 91 (14.7%) patients with permanent stenting, and 211 (34.1) patients with tirofiban administration, however, these intraprocedural options were balanced between groups, except for a higher proportion of permanent stenting in the heparinization group (22.7% vs. 8.6%, *p* < 0.01). Patients underwent heparinization during MT had higher NIHSS score and more likely to be smokers and drinkers, more likely to be categorized as cardio-embolic stroke and anterior circulation stroke (ACS). On the other hand, there are significantly more patients in the non-heparinization group ascribed to large artery atherosclerotic stroke and posterior circulation stroke (PCS).

**Table 1:** Baseline characteristics of patients according to heparin use.

**Characteristic**	**All patients** ***n* = 619**	**Heparinization** ***n* = 269**	**Non-heparinization** ***n* = 350**	***P*-value**
Age, mean±SD	63.9 ± 13.7	63.6 ± 14.7	64.1 ± 12.9	0.83
Male, (*n*%)	404 (65.3)	174 (64.7)	230 (65.7)	0.80
Bridging IVT, (*n*%)	190 (30.7)	74 (27.5)	116 (33.1)	0.14
ASPECTS^a^, median (IQR)	8 (7–8)	8 (8–9)	8 (7–8)	0.14
Admission NIHSS, median (IQR)	16 (13–22)	17 (14–22)	16 (11–22)	0.02
OTP time, median (IQR), min	270 (200–367)	268 (205–368)	270 (195–363)	0.85
OTR time, median (IQR), min	371 (290–460)	371 (285–450)	371 (290–473)	0.39
Pre-antiplatelet (*n*%)	137 (22.1)	66 (24.5)	71 (20.3)	0.24
**VASCULAR RISK FACTORS (*****N*****%)**				
Previous ischemic stroke	72 (11.6)	33 (12.3)	39 (11.1)	0.71
Hypertension	334 (54.0)	136 (50.6)	198 (56.6)	0.14
Diabetes mellitus	96 (15.5)	34 (12.6)	62 (17.7)	0.09
Atrial fibrillation	140 (22.6)	66 (24.5)	74 (21.1)	0.33
Moderate to heavy alcohol consumption	257 (41.5)	152 (56.5)	105 (30.0)	<0.01
Previous or current smoker	207 (33.4)	112 (41.6)	95 (27.1)	<0.01
**PATHOGENESIS OF STROKE, (*****N*****%)**				
Large artery atherosclerosis	385 (62.2)	147 (54.7)	238 (68.0)	<0.01
Cardio-embolism	132 (21.3)	77 (28.6)	55 (15.7)	<0.01
Other	102 (16.5)	45 (16.7)	57 (16.3)	0.91
Anterior circulation stroke (*n*%)	471 (76.1)	224 (83.3)	247 (70.6)	<0.01
Posterior circulation stroke (*n*%)	148 (23.9)	45 (16.7)	103 (29.4)	
**INTRAPROCEDURAL OPTIONS (*****N*****%)**				
Catheter aspiration	51 (8.2)	26 (9.7)	25 (7.1)	0.30
Balloon angioplasty	90 (14.5)	43 (16.0)	47 (13.4)	0.42
Permanent stenting	91 (14.7)	61 (22.7)	30 (8.6)	<0.01
Tirofiban administration	211 (34.1)	97 (36.1)	114 (32.6)	0.39

*IQR, interquartile range; IVT, intravenous thrombolysis; ASPECTS, Alberta Stroke Program Early CT Score; NIHSS, National Institutes of Health Stroke Scale score; OTP time, onset to puncture time; OTR time, onset to recanalization time; MT, mechanical thrombectomy*.

a *ASPECTS score for anterior circulation stroke only*.

All safety and efficacy outcomes were shown in Table 2 for the entire cohort, heparinization and non-heparinization group. Table 3 depicted the associations of baseline characteristics with sICH and poor outcome (mRS 3~5) in univariable and multivariable logistic regression analyses.

**Table 2:** Safety and efficacy endpoints of patients grouped by heparinization.

	**ALL PATIENTS (*N* = 619)**							
	**All**	**Heparinization *n* = 269**	**Non-heparinization *n* = 350**	**OR^a^/HR^b^** **(95% CI)**	***P*-value**	**Adjusted OR^a^/HR^b^** **(95% CI)**	**Adjusted *P*-value**	***P* for interaction^c^**
sICH	43 (6.9)	25 (9.3)	18 (5.1)	1.89 (1.01–3.54)	0.05	2.34 (1.17–4.68)	0.02	0.63
Total ICH	96 (15.5)	49 (18.2)	47 (13.4)	1.44 (0.93–2.22)	0.12	1.60 (0.98–2.61)	0.06	0.75
Distal embolization	30 (4.8)	19 (7.1)	11 (3.1)	2.34 (1.10–5.01)	0.04	2.43 (1.05–5.59)	0.04	0.32
Recanalization	536 (86.6)	232 (86.3)	304 (86.9)	0.95 (0.60–1.51)	0.91	0.91 (0.54–1.52)	0.71	0.38
**THREE-MONTH FOLLOW-UP OUTCOME**								
Functional independence	273 (44.1)	107 (39.8)	166 (47.4)	0.73 (0.53–1.01)	0.06	0.61 (0.41–0.90)	0.01	0.68
Mortality	126 (20.4)	52 (19.3)	74 (21.1)	0.89 (0.60–1.33)	0.62	1.14 (0.70–1.86)	0.61	0.09

*OR, odds ratio; HR, hazard ratio; sICH, symptomatic intracranial hemorrhage; Functional independence, mRS 0–2*.

*Adjusted for age, gender, admission NIHSS, smoking, drinking, pathogenesis of stroke (large-artery atherosclerosis and cardio-embolism), infarct location (anterior circulation stroke and posterior circulation stroke), intravenous thrombolysis, rescue tirofiban, operative procedures (permanent stenting)*.

a *OR or Adjusted OR for recanalization, total ICH, sICH, distal embolization and long-term functional independence*.

b *HR or Adjusted HR for long-term mortality*.

c *P for interaction: interaction effect of rescue tirofiban by infarct locations (anterior circulation stroke and posterior circulation stroke) for risk of safety and efficacy endpoints*.

**Table 3:** Univariable and multivariable logistic regression analyses of the associations of heparinization and other baseline characteristics with the probability of sICH and poor outcome following mechanical thrombectomy in patients with emergent large vessel occlusion.

**Variable**	**sICH**				**Poor outcome**			
	**Univariable logistic regression analysis**		**Multivariable logistic regression analysis**		**Univariable logistic regression analysis**		**Multivariable logistic regression analysis**	
	**Odds ratio** **(95% CI)**	***P*-value**	**Odds ratio** **(95% CI)**	***P*-value**	**Odds ratio** **(95% CI)**	***P*-value**	**Odds ratio** **(95% CI)**	***P*-value**
Age	1.0 (0.98–1.03)	0.88	–	–	1.04 (1.03–1.06)	<0.01	1.04 (1.03–1.06)	<0.01
Bridging IVT	1.23 (0.64–2.36)	0.54	–	–	0.53 (0.37–0.75)	<0.01	0.57 (0.39–0.82)	<0.01
Admission NIHSS	1.03 (0.98–1.07)	0.29	–	–	1.12 (1.08–1.15)	<0.01	1.11 (1.07–1.14)	<0.01
**VASCULAR RISK FACTORS**								
Hypertension	0.66 (0.35–1.22)	0.19	–	–	1.31 (0.95–1.80)	0.10	1.08 (0.76–1.54)	0.68
Moderate to heavy alcohol consumption	0.59 (0.30–1.15)	0.12	–	–	0.71 (0.52–0.99)	0.04	0.69 (0.48–0.99)	0.04
Cardio-embolism stroke	1.83 (0.89–3.77)	0.10	1.89 (1.05–3.96)	0.04	0.92 (0.62–1.35)	0.66	–	–
Posterior circulation stroke	0.31 (0.11–0.88)	0.03	0.33 (0.11–0.98)	0.05	1.87 (1.27–2.76)	<0.01	1.84 (1.20–2.83)	<0.01
Heparinization	1.89 (1.01–3.54)	0.04	2.36 (1.19–4.67)	0.01	1.41 (1.02–1.95)	0.03	1.79 (1.23–2.59)	<0.01

*sICH, symptomatic intracranial hemorrhage; CI, confidence interval; IVT, intravenous thrombolysis; NIHSS, National Institutes of Health Stroke Scale*.

### Safety Outcomes

Overall, 43 (6.9%) patients developed sICH within 24 h post-MT, including 25 (9.3%) in heparinization group and 18 (5.1%) in non-heparinization group. Ninety six (15.5%) and 30 (4.8%) patients experienced total ICH and distal embolization, respectively. Patients in heparinization group had significantly higher risk of sICH and distal embolization even after adjustment for potential confounding factors compared with patients in non-heparinization group (9.3 vs. 5.1%, adjusted *p* = 0.02; 7.1 vs. 3.1%, adjusted *p* = 0.04, respectively). However, no significant between-group difference over total ICH was observed (Adjusted *p* = 0.06). Multivariable logistic regression analyses in Table 3 showed that heparinization during MT was an independent predictor for sICH (Odds ratio 2.36 [1.19–4.67], *p* = 0.01) in addition to cardio-embolism stroke and PCS (Odds ratio 1.89 [1.05–3.96], *p* = 0.04; Odds ratio 0.33 [0.11–0.98], *p* = 0.05, respectively).

There were no interaction effects of heparinization during MT by infarction location (ACS or PCS) for the risk of sICH, total ICH and distal embolization (p for interaction = 0.63, 0.75, and 0.32 in the adjusted model, respectively).

### Efficacy Outcomes

Overall, 536 (86.6%) patients achieved successful artery recanalization, including 232 (86.3%) in heparinization group and 304 (86.9%) in non-heparinization group. No significant between-group difference was found over arterial recanalization (adjusted *p* = 0.71).

**Figure 2:**
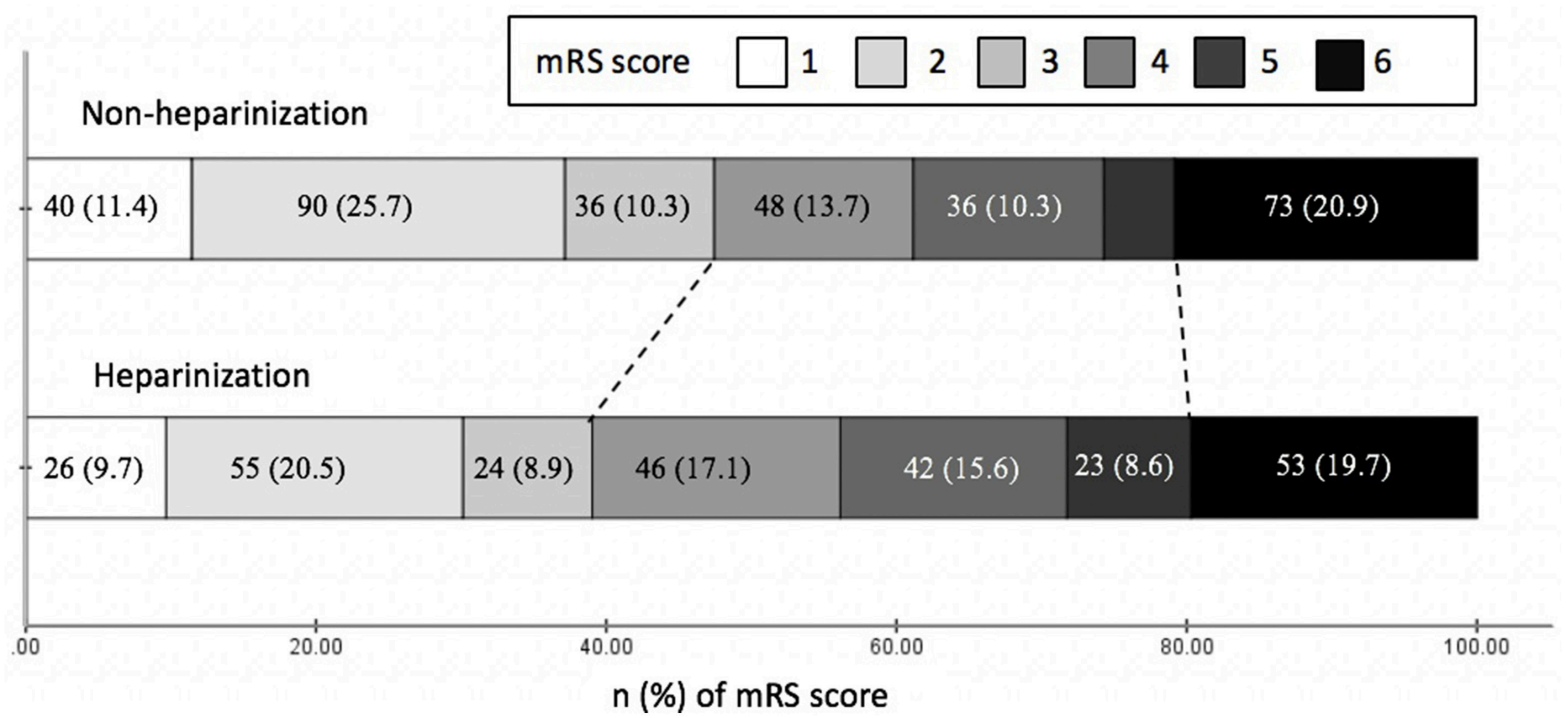
Clinical outcomes at three months by modified Rankin Scale (mRS) of patients with and without heparinization during mechanical thrombectomy.

After 3-month follow-up, 273 (44.1%) patients achieved functional independence and 126 (20.4%) patients died. There were significantly more patients achieved functional independence in non-heparinization group than that in heparinization group (47.4 vs. 39.8%, adjusted *p* = 0.01), and no such between-group difference was observed over mortality (adjusted *p* = 0.61) (Figure 2). Multivariable logistic regression analyses in Table 3 showed that heparinization during MT was an independent predictor for poor outcome [Odds ratio 1.79 [1.23–2.59], *p* < 0.01] besides age [Odds ratio 1.04 [1.03–1.06], *p* < 0.01], bridging IVT [Odds ratio 0.57 [0.39–0.82], *p* < 0.01], admission NIHSS [Odds ratio 1.11 [1.07–1.14], *p* < 0.01], drinking [Odds ratio 0.69 [0.48–0.99], *p* = 0.04], and PCS [Odds ratio 1.84 [1.20–2.83], *p* < 0.01].

There were no interaction effects of heparinization during MT by infarction location (ACS or PCS) for the risk of artery recanalization, functional independence and mortality after 3-month follow-up (p for interaction = 0.38, 0.68, and 0.09 in the adjusted model, respectively).

## Discussion

In this prospective registry study, we found significantly more patients in the heparinization group suffered sICH and distal embolization, and significantly less patients achieved long-term functional independence compared with that in non-heparinization group. Besides, heparinization during MT was an independent predictor for sICH [Odds ratio 2.36 [1.19–4.67], *p* = 0.01] in addition to cardio-embolism stroke and PCS, and was also an independent predictor for poor outcome [Odds ratio 1.79 [1.23–2.59], *p* < 0.01] besides age, bridging IVT, admission NIHSS, drinking and PCS. Accordingly, results may be interpreted as that heparinization during MT should proceed prudently for its probable association with sICH, distal embolization and poor outcome, although more randomized controlled trials are necessary.

Systemic heparinization has already been a mainstay procedure even during conventional endovascular treatment (EVT) for preventing intraprocedural distal embolization or clot extension (15). The primary mechanism of unfractionated heparin is to catalyze antithrombin III-mediated inactivation of thrombin, factors IXa, Xa, XIa, XIIa so as to produce the antithrombotic effects (16). The intention of heparinization during MT is to impede further clot development by its anticoagulant effects and additional inhibition on platelet aggregation. Nonetheless, these potential benefits with heparinization during MT must be considered along with the potential dose-dependent risk of hemorrhagic complications (17). Although AHA/ASA Guideline has recommended for pursuit of MT for AIS but without recommendations on the use of heparin during MT (18). Moreover, no standard protocol for intraprocedural heparin administration heightens the difficulty to evaluate the net benefit of heparinization during MT. Earlier trials on the use of intravenous heparin for AIS including the Trial of ORG 10172 in Acute Stroke Treatment (TOAST) (19) and the International Stroke Trial (IST) (20) showed that intravenous heparin was associated with increased risk of dose-dependent ICH even with state-of-the-art dosing regimen and under rigorous control. As to heparinization during EVT for AIS, the PROACT trial (21), —a randomized controlled trial on intra-arterial recombinant prourokinase—stratified subjects into higher (100 IU/kg bolus followed by 1,000 IU/h for 4 h) and lower (2,000 IU bolus followed by 500 IU/h) heparin dosing group, and indicated an dose-dependent effects of heparin on recanalization and sICH.

However, few studies have focused on heparinization during MT, especially with guideline-recommended newer generation thrombectomy stents or aspiration devices. *Post-hoc* analysis of Multi MERCI (3) and TREVO 2 (5) trial on periprocedural heparinization reported a relative improvement over long-term functional independence, although one of them suggested an increased risk of sICH. Besides, Farook et al. (4) indicated the safety profile of intraprocedural heparinization through a small-sized retrospective study through an interventional stroke database from 2009 to 2012. In general, given the small sample size and heterogeneous treatment protocol of these preliminary studies of MT, caution is compulsory when generalizing from these conclusions. In this study, we found that heparinization during MT was significantly associated with increased risk of sICH and long-term poor functional outcome. Postulated mechanisms accounted for this discrepancy include the following. First, we adopted the state-of-the-art thrombectomy or aspiration devices such as Solitaire AB/FR (Covidien/ev3, Irvine, CA), Trevo Proview (Stryker, CA) and Penumbra (Alameda, CA), achieving overall as high as 86.6% in recanalization rate. In contrast, the three MT studies above were all conducted before 2012 year with thrombectomy devices of first generation, achieving 55.4~66.7% in recanalization rate even in the heparinization group (3–5). It may be postulated that the benefit of heparin on artery recanalization is limited to situations when artery recanalization is unfavorably achieved with old and low-efficiency thrombectomy devices. In contrast, in the cases of favorable recanalization by newer generation thrombectomy devices, the adverse effects of heparin over hemorrhagic complications emerge and counteract its potential benefit on artery recanalization. Second, subjects in Multi MERCI (22) and TREVO 2 (23) trial underwent MT with rescue bridging IVT, which was defined as failure to artery recanalization with IVT. In contrast we performed MT with direct bridging IVT that IVT was conducted while preparing for MT without observation time for the efficacy of IVT. And results indicated that MT with rescue bridging IVT achieved much higher incidence of sICH (22.4 and 25.2% for heparinization and non-heparinization group) and all ICH (37.5 and 44.4%) than that in our study (sICH: 9.3 and 5.1%, all ICH: 18.2 and 13.4%, respectively) both for the heparinization and non-heparinization group, but no significant differences between heparinization group and non-heparinization group were found in these earlier studies (3, 5). It may be postulated that the adverse effect of heparinization during MT for hemorrhagic complications are more obvious for patients at a lower incidence of ICH, although inconclusive. Last but not the least, several rescue therapies were adopted for improved artery recanalization including balloon angioplasty, permanent stenting, intra-arterial thrombolysis and intra-arterial/intravenous tirofiban administration for recanalization refractory patients. Thus, the intraprocedural heparin may act synergistically with thrombolytics or tirofiban on antithrombosis, which may raise the risk of hemorrhagic complications (24), especially for patients with arterial endothelial injury or delayed recanalization.

Besides, we got a novel finding that heparinization during MT was associated with increased risk of distal embolization. Distal embolization has been reported to be due to clot fragments during thrombectomy procedures and is predictive of worse clinical outcomes (25). We found similar overall rate (4.8%) of distal embolization as previous studies reported (14, 26). But 7.1% of patients in heparinization group developed distal embolization compared with that in the non-heparinization group, which we postulated that the softening effects on the thrombus derived from the antithrombotic effects of heparin may potentially generate clot fragment and cause distal embolization.

The main strength of this study lies in its prospective, large-sample and multi-centric design, which strengthens the generalizability of the results. However, there are several limitations. First and foremost, heparinization during MT was performed empirically at the discretion of the operator according to local protocols instead of randomization strategies, which would cause selection bias and add to the difficulty in reflecting the independent effect of heparinization. Second, heparin produces dose-dependent antithrombotic effect and risk of hemorrhagic complications (17). In this study, we performed heparin administration within a certain dosage range instead of a dose-stratification study, which may mute the therapeutic effects of heparinization at a particular dose. Thus, further randomized dose-escalation studies are needed to determine the safety and efficacy of heparinization. Third, we didn't monitor the activated clotting time (ACT) to evaluate the degree of heparinization. In addition, all subjects were Chinese, which possess high prevalence of intracranial atherosclerosis (ICAS) (27). Thus, the results from our study may not be generalizable to the overall population.

In conclusion, this prospective cohort study indicated that heparinization during MT was associated with increased risk of safety outcomes over sICH and distal embolization, as well as efficacy outcomes over long-term poor functional outcomes. Accordingly, it follows that heparinization during MT should not be advocated, although further randomized controlled studies are needed.

## Data Availability

The raw data supporting the conclusions of this manuscript will be made available by the authors, without undue reservation, to any qualified researcher.

## Author Contributions

MY and XH study concept and design, data analysis and interpretation, manuscript drafting. FG, AW, and NM acquisition of data, study supervision and coordination. DL study concept, interpretation of the data, and comments on the drafts. YW and ZM study concept and design, obtaining funding, study supervision and coordination. YW and ZM had full access to all the data and take responsibility for the integrity of the data and the accuracy of the data analysis.

### Conflict of Interest Statement

The authors declare that the research was conducted in the absence of any commercial or financial relationships that could be construed as a potential conflict of interest.
